# Examining the effects of second-and third-trimester gestational weight gain rates on the perinatal outcomes among Chinese twin pregnancies: a retrospective cohort study

**DOI:** 10.1186/s12884-022-04467-8

**Published:** 2022-02-19

**Authors:** Li-hua Lin, Yi-lin Weng, Ying-ying Lin, Xiu-xian Huang, Yang Lin, Xiao yan Xiu, Jian-ying Yan, Juan Lin

**Affiliations:** 1 Department of Healthcare, Fujian Maternity and Child Health Hospital, Affiliated Hospital of Fujian Medical University, Fujian Province 350001 Fuzhou, People’s Republic of China; 2 Department of Obstetrics, Fujian Obstetrics and Gynecology Hospital, Fujian Province 350001 Fuzhou, People’s Republic of China; 3 Department of Obstetrics, Fujian Maternity and Child Health Hospital, Affiliated Hospital of Fujian Medical University, Fujian Province 350001 Fuzhou, People’s Republic of China; 4 Department of Health Education, Fujian Maternity and Child Health Hospital, Affiliated Hospital of Fujian Medical University, Fujian Province 350001 Fuzhou, People’s Republic of China

**Keywords:** Twin Pregnancies, Gestational Weight Gain, Perinatal Outcome

## Abstract

**Background:**

This paper investigated how second- and third-trimester gestational weight gain relates to perinatal outcomes among normal weight women with twin pregnancies in Fujian, China.

**Methods:**

A retrospective study examining the medical records of 931 normal weight twin-pregnant women was conducted in Fujian Maternity and Child Health Hospital from 2014 to 2018.The 2^nd^ and 3^rd^trimester weekly weight gain rates were calculated, and women were categorized as gaining below, within, or above the 2009 Institute of Medicine (IOM) recommended rates. The association between the trimester-specific weight gain rate and perinatal outcome was determined by traditional regression analysis among groups.

**Results:**

A total of 25.9%, 19.8% and 54.3% of women had rates of weight gain across the 2^nd^ and 3^rd^ trimesters less than, greater than or within the recommended rates respectively. Multivariate logistic regression analysis showed that weight gain greater than the recommended rate in the 2^nd^ trimester was associated with a decreased risk of preeclampsia (aOR:0.489,95%CI:0.289 ~ 0.974). Weight gain less than the recommended rate of weight gain in the 3^rd^ trimester was associated with increased risks of premature delivery(aOR:2.079, 95%CI:1.467 ~ 2.968), gestational diabetes mellitus (aOR: 2.048, 95%CI:1.411 ~ 2.971), intrahepatic cholestasis syndrome (aOR:3.015,95%CI: 1.058 ~ 8.587), pre-labour rupture of membrane (aOR: 1.708,95%CI: 1.169 ~ 2.493), average twin birth weight < 2500 g(aOR:1.532,95%CI: 1.125 ~ 2.084) and neonatal respiratory distress syndrome (aOR:4.934,95%CI:1.626 ~ 15.083) and was associated with decreased risks of caesarean section (aOR:0.589,95%CI:0.386 ~ 0.898) and preeclampsia (aOR:0.471, 95%CI:0.274 ~ 0.808). In addition, weight gain greater than the recommended rate of weight gain in the 3^rd^ trimester was associated with increased risks of premature delivery (aOR:1.589,95%CI:1.428 ~ 2.951) and gestational hypertension (aOR:2.137,95% CI:1.034 ~ 4.415) as well as preeclampsia (aOR:2.246, 95%CI:1.462 ~ 3.452). The stratified analysis of weight gain in the 3^rd^ trimester showed that there was no significant difference in the incidence of adverse pregnancy outcomes compared to the 2^nd^ trimester weight gain groups.

**Conclusions:**

While this study showed that a gestational weight gain rate above or below the recommendation in the 3^rd^ trimester was associated with some adverse maternal and neonatal outcomes, further prospective and multicentre studies are required to explore alternate ranges of gestational weight gain rates in twin pregnancies.

## Background

Gestational weight gain (GWG) is an important indicator for assessing the nutritional status of pregnant women and is closely related to foetal growth and health [1, 2]. Excessive or inadequate weight gain during pregnancy can affect the short- and long-term health of the mother and foetus. Both excessive and inadequate weight gain during pregnancy are associated with adverse pregnancy outcomes. Studies have shown that excessive GWG increases the risk of hypertensive disorders of pregnancy, gestational diabetes, macrosomia, large for gestational age, and caesarean section and even affects the future growth trajectory of the child, with consequences including childhood and adolescent obesity, while inadequate weight gain during pregnancy increases the risk of miscarriage, premature delivery, foetal growth restriction and stillbirth [3–6]. The Institute of Medicine (IOM) published guidelines on appropriate weight gain ranges during pregnancy in 1990 and revised them in 2009 to establish appropriate weight gain ranges for singleton pregnancies and temporary weight gain ranges for twins based on pre-pregnancy body mass index [1]. The revised guidelines included recommended rates of 2^nd^ and 3^rd^trimester weight gain in pounds per week for singleton pregnancies but not for twin pregnancies. Compared with singleton pregnancies, twin pregnancies have attracted much attention due to their more complex physiological characteristics and higher incidence of adverse pregnancy outcomes. Numerous studies have been devoted to the study of GWG in twin pregnancies to explore the applicability of the IOM recommendations for twin pregnancies in China, but no consensus has been reached. GWG and adverse events in twin pregnancies are often confounded by the length of gestation. Most studies mainly focus on total weight gain throughout pregnancy, while relatively few studies have been devoted to the relationship between weight gain rates in different trimesters and pregnancy outcomes. It has been clinically observed that GWG is not linear throughout pregnancy, with slower increases in the 1^st^ trimester and a more uniform rate in the 2^nd^ and 3^rd^ trimesters [7]. Therefore, this study aimed to assess the effect of second- and third-trimester rates of GWG on maternal and neonatal outcomes using the 2009 revised IOM guidelines for twin pregnancies. Due to sample size limitations, only the women with twin pregnancies and a normal pre-pregnancy body mass index (BMI) were analysed in this study.

## Methods

### Study population

We retrospectively reviewed the medical records of women with normal pre-pregnancy BMIs who received perinatal care and delivered twins at the Fujian Maternity and Child Health Hospital between 2014 and 2018. The inclusion criteria were as follows: (1) maternal age between 18 and 45 years; (2) both twins were alive at birth; (3) complete medical records with pre-pregnancy weight, height, twin birth weights, gestational age, and weight measurements within 1 week of delivery; and (4) the mothers had no chronic diseases, such as diabetes or hypertension diagnosed before pregnancy. We excluded women with twin pregnancies that had been reduced from multiple pregnancies or those whose weight records were missing. The use of weight gain rates also allowed for the inclusion of women who gave birth prematurely.

As this was a retrospective study that did not involve interactions with the women included in the study or data that could be used to identify them, the Ethics Committee of the Fujian Maternity and Child Health Hospital approved it and waived the requirement for informed consent.

### Information collection and definitions

Patient data of demographic information and maternal characteristics was extracted from medical records. Data collection process and definitions have been described in detail elsewhere [8]. Pre-pregnancy BMI was calculated as [pre-pregnancy weight (kg)]/height^2^(m^2^)]. Due to the sample size limitation, only normal weight pregnant women (BMI 18.5–24.9 kg/m^2^) were included in this study. Delivery weight was defined as the weight at delivery or the last measured weight within 1 week before delivery. The IOM guidelines include recommended rates for weight gain in the 2^nd^ and 3^rd^ trimesters per week for singleton pregnancies for underweight, normal- weight,overweight and obese women are 0.44 – 0.58 kg, 0.35–0.50 kg, 0.23–0.33 kg and 0.17–0.27 kg, respectively, whereas no weekly weight gain rate recommendation have been made for twin pregnancies [1]. In addition, the weekly rate of weight gain in the 2^nd^ and 3^rd^ trimesters for singleton pregnancies was consistent with IOM guidelines, so we assumed that it was the same as that for twin pregnancies. When assuming the same weekly rate of weight gain in the 2^nd^ and 3^rd^ trimesters, we estimated the weekly rate of the 2^nd^ and 3^rd^trimesters using linear interpolation to calculate the weight at 28 weeks and 29 weeks by using the closest weight measured before or after 28 weeks and 29 weeks. The rates of total 2^nd^ and 3^rd^ trimester weight gain were calculated according to the following formula: (pregnancy weight at delivery—weight in the 13^th^ week of pregnancy)/(gestation at delivery—13 weeks). The rate of 2^nd^ trimester weight gain was calculated as the difference between the mother’s weight at 28 weeks and at 13 weeks divided by the gestational age interval, and the rate of 3^rd^ trimester weight gain in pregnancy was calculated as the difference between the mother’s weight at delivery and at 29 weeks divided by the gestational age interval. According to the IOM guidelines [1], the recommended total GWG for twin pregnancies is 16.8–24.5 kg for normal weight women who deliver twins at term (37–42 weeks of gestation) with an average new-born birth weight ≥ 2500 g, and the average weight gain in 1^st^ trimester is 3.6 kg. The 1^st^ trimester was defined as the first 13 weeks of pregnancy. Referring to the relevant literature [9] and taking into account the actual gestational age at delivery in the included sample, it was assumed that a term twin pregnancy was 38 weeks. On this basis, we calculated and compared the 2^nd^ and 3^rd^ trimester weekly rates of weight gain according to the IOM guidelines and the actual value for each woman, and accordingly, women were categorized into groups with less than, within and greater than the recommended rate of second^−^or 3^rd^ trimester weight gain according to the IOM guidelines.

Perinatal outcomes in this study included the following maternal outcomes: gestational hypertension, defined as blood pressure ≥ 140/90 mmHg that occurred after 20 weeks gestation but without proteinuria; preeclampsia, diagnosed upon the development of blood pressure ≥ 140/90 mmHg and proteinuria ≥ 300 mg/24 h after 20 weeks gestation; gestational diabetes mellitus, reported after a 75 g oral glucose tolerance test (OGTT) at 24–28 weeks gestation when at least one of the abnormal blood glucose levels was observed(fasting blood glucose level ≥ 5.1 mmol/L, a 1-h blood glucose level ≥ 10.0 mmol/L, or a 2-h blood glucose level ≥ 8.5 mmol/L);pre-labour rupture of membranes, referred to the rupture of membranes prior to labour; intrahepatic cholestasis of pregnancy, a common pregnancy-specific liver disorder associated with elevated bile acids, pruritus, abnormal liver function tests, and an increased incidence of adverse foetal outcomes;postpartum haemorrhage, defined as bleeding of more than 500 ml 24 h after vaginal delivery or more than 1000 ml 24 h after caesarean delivery; and other pregnancy outcomes including caesarean delivery, premature delivery < 37 weeks, low birth weight (LBW, average twin birth weight < 2500 g), small for gestational age(SGA), inconsistent birth weight of twins (twin birth weight difference ≥ 20%), and neonatal respiratory distress syndrome. SGA was defined as a birth weight less than the 10th percentile according to twin fetal growth curves [10, 11]. Neonatal respiratory distress syndrome was defined as an infant displaying an Apgar score ≤ 7 at 1 or 5 min after birth [12].

Additional maternal demographic characteristics and perinatal factors were available, including maternal age and height, level of maternal education, gravidity, nulliparity, chorionicity, and infant sex. Nulliparity was defined as no prior births greater than 20 weeks of gestation. Chorionic membrane included monochorionic and dichorionic membranes.

### Statistical analyses

Baseline characteristics were compared across groups of 2^nd^ and 3^rd^trimester weekly rates of GWG: less than, within and greater than the IOM guidelines. Continuous variables are described as the mean ± standard deviation (SD) and were compared with analysis of variance (ANOVA) or the Kruskal–Wallis test. Categorical variables are described as frequencies and were compared with a chi-square test or Fisher’s exact test. We performed multiple comparisons (Scheffe method) to assess the differences between each of the groups if the results of ANOVA or chi-squared analysis were statistically significant. Logistic regression analysis was applied to estimate the independent effect of the 2^nd^ or 3^rd^ trimester weekly rate of GWG on perinatal outcomes. Adjusted odds ratios (OR) and 95% confidence intervals(95%CI) were used to report the effect estimates. The multivariable analysis was adjusted for maternal age, gestational age, infant sex, pre-pregnancy BMI, nulliparity, education level, and average infant weight.However, the average infant weight gain was not adjusted for in the weight related outcomes ie average birth weight,SGA and weight discordance ≥ 20% in multivariable analysis. All the effect estimates were presented graphically as forest plots. *P* < 0.05 was considered a statistically significant difference. Statistical analysis was performed using STATA15.0.

## Results

**Fig. 1:**
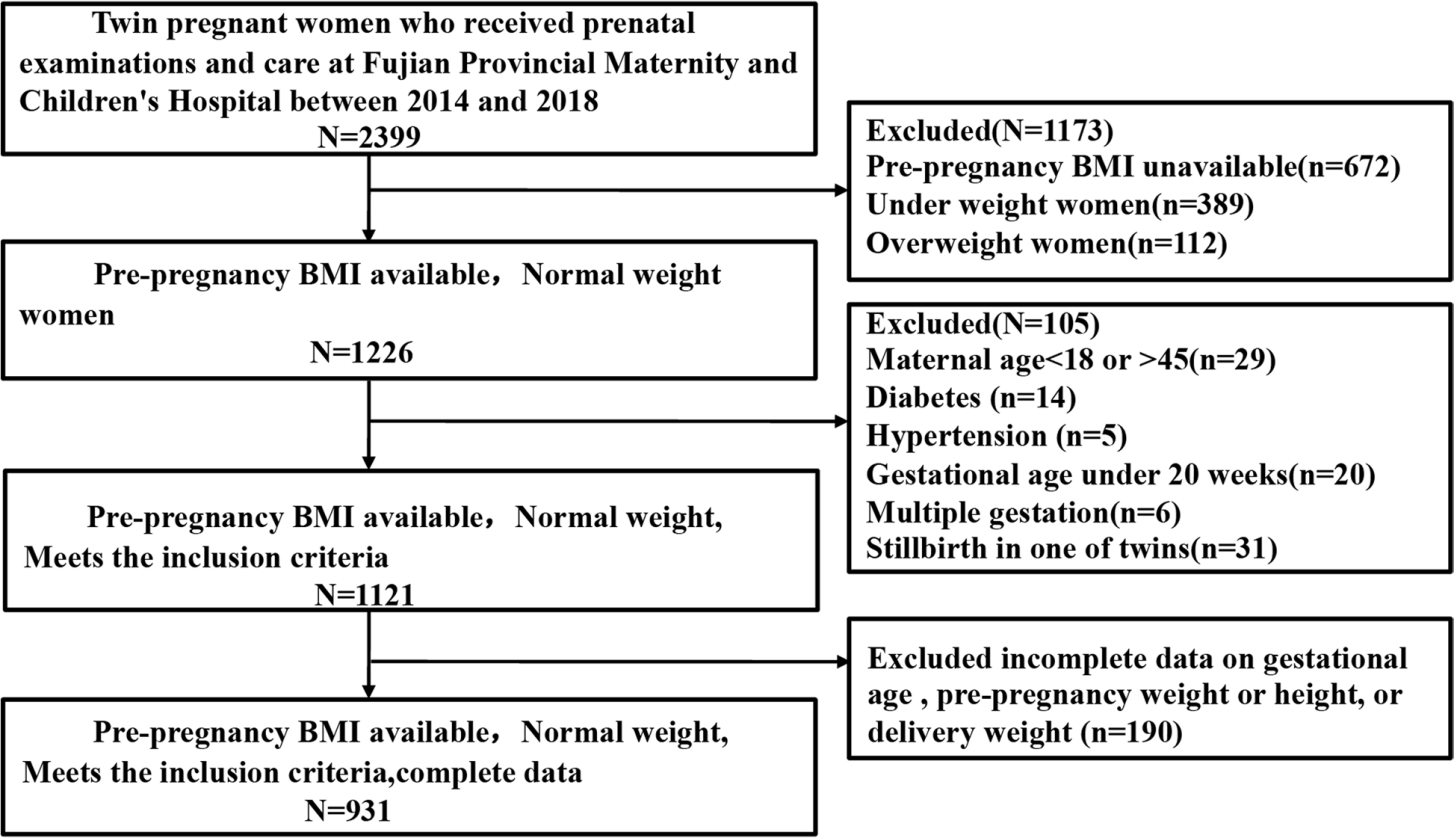
Flowchart of Subject selection

A total of 2399 pregnant women received perinatal care and delivered twins at the Fujian Maternity and Child Health Hospital during the study period from 2014 to 2018. Of these, there were 1226 pregnant women with normal weight. We excluded 29 women as a result of maternal age < 18 or > 45 years, pre-existing diabetes (*n* = 14), chronic hypertension (*n* = 5), gestational age under 20 weeks (*n* = 20),multiple gestations (*n* = 6), stillbirth of one twin (*n* = 31), and missing records of gestational age, pre-pregnancy weight or height, or delivery weight (*n* = 190). There were 931 pregnant women and 1862 infants included (Fig. 1).

The majority of twin-pregnant women in this study had 2^nd^ and 3^rd^trimester rates of weight gain within the IOM recommendations. That is, 54.3% of women had estimated 2^nd^ and 3^rd^ trimester rates of weight gain meeting the recommended rates, 25.9% of women had weight gain less than the recommended rates, and 19.8% of women had rates greater than the recommended rates. Table 1 shows the differences in the baseline characteristics among the three groups. The average maternal age was 30.1 ± 4.3 years, pre-pregnancy BMI was 21.1 ± 1.6, height was 160.4 ± 5.1 cm, and the mean number of years of education was 12.6 ± 3.3 years. Significant differences were found for age, pre-pregnancy BMI, height, and years of education among women with weekly rates of GWG in the 2^nd^ and 3^rd^ trimesters less than, within and greater than recommended rates (*P* < 0.05). There were no significant differences among the three groups in the proportions of nulliparity, monochorionic membrane or infant sex. The gestational age and average birth weight of the infants were significantly different between any two groups(*P* < 0.001). Women who were excluded from this study due to missing information had similar baseline characteristics as those included (data not shown).

**Table 1:** Baseline characteristics according to the second and third trimester weight gain rate

	All participants (*N* = 931)	Within recommendations (*N* = 506, 54.3%)	Less than recommendations (*N* = 241, 25.9%)	Greater than recommendations (*N* = 184, 19.8%)	*p*^a^
Maternal age(years)	30.1 ± 4.3	30.3 ± 4.2	31.2 ± 4.4	28.3 ± 3.9	< 0.001^b^
Prepregnancy BMI	21.1 ± 1.6	21.0 ± 1.6	21.5 ± 1.6	20.9 ± 1.6	< 0.001
Maternal height(cm)	160.4 ± 5.1	160.3 ± 5.0	159.6 ± 4.8	161.8 ± 5.3	< 0.001
Education degree(years)	12.6 ± 3.3	12.6 ± 3.2	12.9 ± 3.6	12.1 ± 3.0	0.028
Prepregnancy weight(kg)	54.4 ± 5.3	54.0 ± 5.3	54.8 ± 5.4	54.7 ± 5.3	0.104
Gravidity					0.489
1	403(43.3)	208(41.1)	109(45.2)	86(46.7)	
2	253(27.2)	148(29.2)	58(24.1)	47(25.5)	
≥ 3	275(29.5)	150(16.1)	74(30.7)	51(27.7)	
Nulliparity	588(63.2)	307(60.7)	152(63.1)	129(70.1)	0.076
Monochorionic membrane	236(25.3)	129(25.5)	51(21.2)	56(30.4)	0.093
Infant sex					0.598
Both boys	363(39.0)	197(38.9)	102(42.3)	64(34.8)	
Both girls	305(32.8)	169(33.4)	73(30.3)	63(34.2)	
Boy + girl	263(28.2)	140(27.7)	66(27.4)	57(31.0)	
Gestational age at delivery (week)	36.2 ± 2.0	36.4 ± 1.7	35.5 ± 2.6	36.4 ± 1.6	< 0.001
Average birth weight(g)	2506.5 ± 451.8	2533.2 ± 404.0	2385.8 ± 553.6	2591.1 ± 396.0	< 0.001^b^

Date are shown as Mean ± SD for continuous variables and N(%) for categorical variables

^a^*P* value were calculated with the variance (ANOVA) or the Kruskal–Wallis test for continuous variables and with the chi-square test for categorical variables

^b^*P* value is statistically significant between any two groups

### Perinatal outcomes according to IOM classification of weight gain rate in the second trimester

**Fig. 2:**
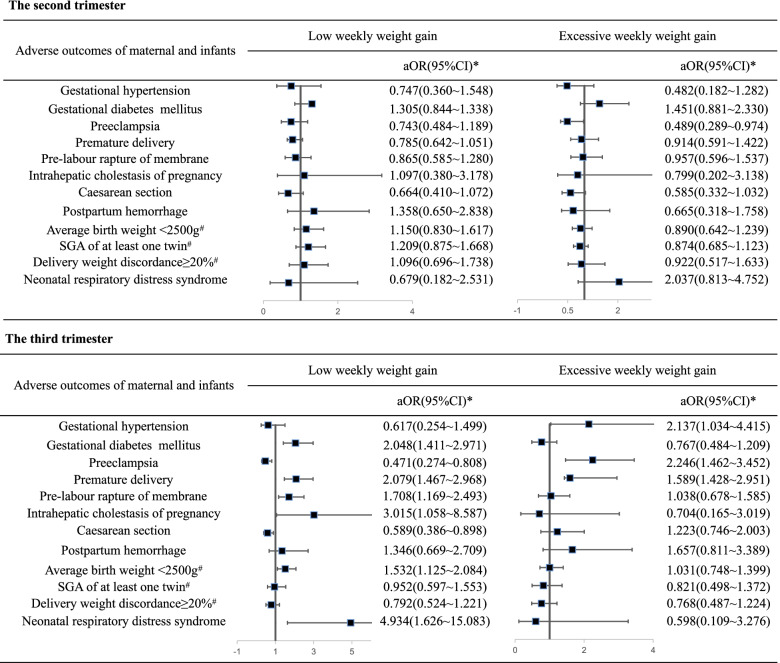
Forest plot of association between perinatal outcomes and second and third trimester weight gain rates. SGA: small for gestation; aOR:adjusted odds ratio; CI:confidence interval; *adjusted for maternal age, gestational age, infant sex, pre-pregnancy body mass index, nulliparity, education level and average infant weight;^#^adjusted for maternal age, gestational age, infant sex, pre-pregnancy body mass index, nulliparity, education level

According to the IOM recommendations, 22.7% and 24.0% of pregnant women gained weight at rates less than and greater than the recommendations in the 2^nd^ trimester, respectively. After controlling for potential confounders in multivariable logistic regression models, weight gain greater than the recommended 2^nd^ trimester rates of weight gain was associated with a decreased risk of preeclampsia (adjusted OR: 0.489, 95% CI: 0.289 ~ 0.974). However, after adjusting for maternal age, gestational age, infant sex, pre-pregnancy BMI, nulliparity, education level and average infant weight, we did not find evidence of an interaction between rates of weight gain less than the recommended 2^nd^ trimester rates and maternal and neonatal outcomes (shown in Fig. 2).

### Perinatal outcomes according to IOM classification of weight gain rate in the third trimester

In the 3^rd^ trimester, 33.2% of the pregnant women gained weight at rates below the recommendation. After controlling for potential confounders in multivariable logistic regression models, they had a higher odds of gestational diabetes mellitus (adjusted OR:2.048, 95% CI: 1.411 ~ 2.971) than pregnant women who gained weight at rates within the recommendations. They also had higher odds of intrahepatic cholestasis syndrome (adjusted OR: 3.015, 95% CI: 1.058 ~ 8.587) and pre-labour rupture of membranes (adjusted OR: 1.708, 95% CI: 1.169 ~ 2.493) and decreased odds of caesarean section (adjusted OR: 0.589, 95% CI: 0.386 ~ 0.898) and preeclampsia (adjusted OR: 0.471, 95% CI: 0.274 ~ 0.808). For neonatal outcomes, we found increased odds for premature delivery (adjusted OR:2.079, 95% CI: 1.467 ~ 2.968), and average twin birth weight < 2500 g(adjusted OR: 1.532, 95% CI: 1.125 ~ 2.084) as well as neonatal respiratory distress syndrome (adjusted OR:4.934, 95% CI:1.626 ~ 15.083).

For the 28.9% of pregnant women who gained weight at rates greater than the recommendations, the adjusted odds of gestational hypertension (adjusted OR = 2.137, 95% CI: 1.034 ~ 4.415), premature delivery (adjusted OR: 1.589, 95% CI: 1.428 ~ 2.951) and preeclampsia (adjusted OR: 2.246, 95%CI: 1.462 ~ 3.452) were significantly different and higher than those among women who gained weight at rates within the recommendations. Other perinatal outcomes did not differ in women who gained weight at rates greater than the guidelines in the 3^rd^trimester compared to those who gained weight within the guidelines (shown in Fig. 2).

### Impact of weight gain rate in second trimester on weight gain rate in third trimester

To evaluate the adverse pregnancy outcomes related to weight gain during second trimester in women with weight gain above or below IOM recommendations in third trimester, we stratified 309 pregnant women who gained weight at rates less than the recommendations in the 3^rd^ trimester (referred to as “T3L”), of whom in the 2^nd^trimester, 53 pregnant women gained weight at rates greater than the recommendations (referred to as “T2H”),156 pregnant women gained weight at rates within recommendations (referred to as “T2N”), and 100 pregnant women gained weight at rates less than the recommendations (referred to as “T2L”). The results showed no statistically significant difference in the incidence of perinatal outcomes between the three groups (*P* > 0.05). Similarly, of 269 pregnant women who gained weight at rates greater than the recommendations in the 3^rd^trimester (referred to as “T3H”), the incidence of pregnancy outcomes were computed based on the classification of weight gain rates in the 2^nd^ trimester, but no statistically significant difference was found among the three groups (all *P*-values > 0.05) (Table 2).

**Table2:** Impact of weight gain rate in second trimester on weight gain rate in third trimester

Adverse outcomes of maternal and infants	T3L				T3H			
	T2N (*N* = 156)	T2L (*N* = 100)	T2H (*N* = 53)	*P1*	T2N (*N* = 141)	T2L (*N* = 34)	T2H (*N* = 94)	*P2*
Gestational hypertension	2(1.3)	4(4.0)	2(3.8)	0.343	13(9.2)	1(2.9)	5(5.3)	0.286
Gestational diabetes mellitus	49(31.4)	36(36.0)	14(26.4)	0.468	19(13.5)	8(23.5)	8(8.5)	0.081
Pre-eclampsia	12(7.7)	3(3.0)	7(13.2)	0.060	36(25.5)	9(26.5)	18(19.1)	0.476
Premature delivery	78(50.0)	47(47.0)	30(56.6)	0.527	66(46.8)	17(50.0)	48(51.1)	0.804
Pre-labour rapture of membrane	39(25.0)	25(25.0)	17(32.1)	0.567	23(16.3)	6(17.6)	18(19.1)	0.854
Intrahepatic cholestasis of pregnancy	8(5.1)	2(2.0)	3(5.7)	0.404	2(1.4)	0(0.0)	1(1.1)	0.646
Caesarean section	122(78.2)	82(82.0)	44(83.0)	0.650	123(87.2)	29(85.3)	86(91.5)	0.489
Postpartum hemorrhage	13(8.3)	3(3.0)	2(3.8)	0.161	14(9.9)	1(2.9)	3(3.2)	0.071
Average birth weight < 2500 g	74(47.4)	49(49.0)	29(54.7)	0.657	61(43.3)	13(38.2)	35(37.2)	0.627
SGA of at least one twin	60(38.5)	49(49.0)	17(32.1)	0.090	61(43.3)	14(41.2)	30(31.9)	0.209
Delivery weight discordance ≥ 20%	18(11.50)	14(14.0)	8(15.1)	0.745	22(15.6)	3(8.8)	9(9.6)	0.299
Neonatal respiratory distress syndrome	8(5.1)	3(3.0)	6(11.3)	0.095	0(0.0)	0(0.0)	2(2.1)	NA

Date are shown as N(%)

P1 and P2 value were calculated with chi-square test or Fisher’s exact test as appropriate

T3L:gained weight at rates less than the recommendations in 3^rd^ trimester;T3H:gained weight at rates greater than the recommendations in 3^rd^ trimester;T2N:gained weight at rates within the recommendations in 2^nd^ trimester;T2L:gained weight at rates less than the recommendations in 2^nd^ trimester;T2H:gained weight at rates greater than the recommendations in 2^nd^ trimester;SGA:small for gestation;NA: The low incidence of neonatal respiratory distress syndrome prohibited their exploration with chi-square test or Fisher’s exact test

## Discussion

It has been reported that the incidence of twin pregnancies has been increasing worldwide with the changing concept of fertility and the development and popularization of assisted reproductive technology. The fact that twin pregnancies are more complex physiological processes than singleton pregnancies [13], coupled with the importance of the impact of GWG on perinatal outcomes, makes it important to examine the recommendations of the IOM guidelines on pregnancy outcomes. Previous studies have focused mainly on the effect of total GWG on pregnancy outcomes, in which gestational age may be a major confounding factor. One study found that total GWG was positively correlated with pregnancy length in all BMI groups [14]. That is, women with shorter gestational ages had a shorter timeframe to gain weight and may suffer adverse pregnancy outcomes such as preterm birth, which may lead to false correlations between GWG and pregnancy outcomes [15]. To this end, we introduced weight gain rates as study variables to evaluate the associations between GWG rates in the 2^nd^ and 3^rd^trimesters and perinatal outcomes among Chinese twin gestations. The 2009 IOM recommended an appropriate weight gain rate for singleton pregnancies in the 2^nd^ and 3^rd^trimesters of pregnancy, but there was no clear recommendation for twin pregnancies, so our study was of some value for the recommendation of weight gain rates for twin pregnancies in the 2^nd^ and 3^rd^trimesters of pregnancy.

In this retrospective study, the findings showed that maternal and neonatal outcomes were affected by the rate of 2^nd^ and 3^rd^ trimester weight gain. We found that a weight gain rate greater than the recommendations in the 2^nd^ trimester was associated with a decreased risk of preeclampsia, while the weight gain rate in the 3^rd^ trimester directly affected the incidence of adverse pregnancy outcomes. On the one hand, a weight gain rate in the 3^rd^ trimester below the recommendations was associated with significant increases in gestational diabetes mellitus, intrahepatic cholestasis syndrome, pre-labour rupture of membranes, average twin birth weight < 2500 g, neonatal respiratory distress syndrome and a lower rate of caesarean section and preeclampsia. On the other hand, a weight gain rate in the 3^rd^ trimester greater than the recommendations was associated with a higher rate of gestational hypertension and premature delivery and preeclampsia. Previous studies have demonstrated that inadequate weight gain during pregnancy was a risk factor for preterm birth, and our results suggested that a low or high weight gain rate in the 3^rd^ trimester was related to an increased risk of preterm birth. The results of the study by Carnero A M et al. [16] and Shamshirsaz AA et al. [17] also showed that both very low and very high weight gain rates were associated with an increased incidence of premature delivery [16]. This finding may also be a reason why many international studies are now questioning the current IOM guidelines for twin pregnancies. Notably, in our study, we found that a low weight gain rate in the 3^rd^ trimester was associated with an increased incidence of gestational diabetes mellitus, which may be contrary to clinical findings. The conflicting results could be due to medical nutritional therapy guidance and intervention by obstetricians based on their experience in managing the nutritional status of pregnant women who were gaining too much weight or who had been diagnosed with gestational diabetes mellitus. These pregnant women adhered to a strict diet and increased their exercise habits, ultimately resulting in a low weight gain rate in the 3^rd^ trimester or even weight gain rates below the guideline recommended range. Conversely, women who were not diagnosed with gestational diabetes mellitus may be less likely to follow a diet or exercise programme, which could lead to more weight gain. This result was similar to the findings of domestic and foreign academics who noted decreased odds of gestational diabetes mellitus in women with excessive rates of weight gain and increased odds of gestational diabetes mellitus in women with suboptimal rates of weight gain in the 2^nd^ trimester and 3^rd^ trimester, due to the nutritional counselling and dietary adjustments that occur after such a diagnosis and the consequent effect on gestational weight gain [18, 19]. In addition, it has been reported that excessive weight gain in the 1^st^ trimester was related to an increased risk of gestational diabetes mellitus, regardless of pre-pregnancy weight [20]. We did not consider weight gain in the 1^st^ trimester, which may lead to an inconsistency between our findings and those of some studies [21].These inconsistencies also suggest that we should not neglect weight management in the 1^st^ trimester of pregnancy.

In this study, we found a higher incidence of neonatal respiratory distress syndrome, average twin birth weight < 2500 g and intrahepatic cholestasis syndrome in pregnant women whose weight gain rate in the 3^rd^ trimester was below the recommendations. We considered that neonatal respiratory distress syndrome may be a secondary association with preterm birth. There are few studies on the symptoms of intrahepatic cholestasis and gestational weight gain, and our results suggested a statistical association; however, the causal association may need to be fully understood, as it has been shown that intrahepatic cholestasis leads to a loss of appetite and nausea, which may have an impact on gestational weight gain, implying that cholestasis may be the cause of lower rates of weight gain rather than lower rates of GWG being the cause of cholestasis. Further studies should be conducted with detailed data on GWG and the timing of signs or symptoms to understand the cause and consequence [22].

For gestational hypertensive disorders, our findings showed that a weight gain rate greater than the recommendation was associated with lower odds of preeclampsia in the 2^nd^ trimester and higher odds of gestational hypertension and preeclampsia in the 3^rd^ trimester. Luke B’s research showed no difference in the weight gain rate in the first half of pregnancy between pregnant women diagnosed with preeclampsia and those without preeclampsia in the second half of pregnancy [23]. The reason for these inconsistencies might be our limited sample size and the limitations of a single-centre study. Domestic and foreign academics have also not reached consensus on the relationship between weight gain in twin pregnancies and hypertensive disorders of pregnancy due to confusion regarding whether weight gain was the cause of or the result of preeclampsia. It may be that pregnancy comorbidities may affect gestational weight gain [14, 24, 25]; for example, gestational diabetes and preeclampsia affect total weight gain during pregnancy [26, 27].

Maternal weight gain in the second half of pregnancy was mainly associated with an increase in foetal weight and placental factors and an increase in maternal body weight and was most closely related to the growth and development of the foetus, especially in the 2^nd^ trimester [28]. In this study, weight gain in the 2^nd^ trimester had little effect on perinatal outcomes and no statistically significant difference was found on perinatal outcomes when stratifying 3^rd^ trimester weekly weight gain less than or greater than the IOM recommendations by weekly weight gain greater than, within or less than IOM recommendations in the 2^nd^ trimester. The finding may be explained as follows: first, by the difference in study population as we included women with a normal pre-pregnancy body mass index due to sample size limitations; second, our use of an assumed average weight gain in the 1^st^ trimester may have resulted in misclassification of exposure, biasing associations toward the null; and third, by other differences that may have been due to the more complex physiological changes that occur in twin pregnancy, which cannot be explained reasonably at present, as further exploration is needed to clarify the mechanisms of physiological changes in twin pregnancy. Even so, physicians should be encouraged to address weight gain throughout the pregnancy and intervene in cases where weight gain falls outside of recommendations.

There were some limitations in this study. First, it was a retrospective study, and it was impossible to consider all potential confounding variables, such as the use of assisted reproductive technology, race/ethnicity of pregnant women, and tobacco use, which may lead to confounding bias. The study data were drawn from data of women with twin pregnancies receiving perinatal care at one hospital, possibly leading to poor representation of the general population. Second, the limited sample size led to limitations in analysing underweight, overweight, and obese women, and the small sample size might result instatistically significant differences in demographic characteristics. Additionally, as the weight gain in twin pregnancies was not linear [29], we were unable to eliminate the correlation between pregnancy length and total weight gain by calculating the weight gain rates [30]. Therefore, our computational approach may introduce some bias, and thus, our study may not be sufficient to assess the associations of certain outcomes.

Future prospective and multicentre studies are warranted. All pregnant women received similar prenatal care and monitoring, which reduces the possibility of sampling bias and contributes to the generalizability of the results, thus clarifying the trajectory of weight gain in twin pregnancies and making the findings more clinically useful.

## Conclusions

In summary, suboptimal and excessive rates of 2^nd^ and 3^rd^ trimester gestational weight gain were associated with adverse pregnancy outcomes in normal weight women, especially in the 3^rd^ trimester. Our findings are useful to help obstetricians understand the key periods of maternal weight gain and provide clinical recommendations to anticipate perinatal outcomes of twin pregnancies. Further prospective and multicentre studies are required to explore alternate ranges of GWG rates in twin pregnancies to reduce adverse perinatal outcomes.

## Data Availability

The datasets used and/or analysed during the current study are available from the corresponding author on reasonable request.

## Abbreviations

GWG: Gestationalweight gain
BMI: Body mass index
IOM: Institute of Medicine
SD: Standard deviation
OGT: Toral glucose tolerance test
LBW: Low birth weight
SGA: Small for gestation
ANOVA: Analysis of variances
OR: Odds ratio
CI: Confidence interval
